# Is Contrast Medium Osmolality a Causal Factor for Contrast-Induced Nephropathy?

**DOI:** 10.1155/2014/931413

**Published:** 2014-03-31

**Authors:** Andreas M. Bucher, Carlo N. De Cecco, U. Joseph Schoepf, Felix G. Meinel, Aleksander W. Krazinski, James V. Spearman, Andrew D. McQuiston, Rui Wang, Judith Bucher, Thomas J. Vogl, Richard W. Katzberg

**Affiliations:** ^1^Department of Radiology and Radiological Science, Medical University of South Carolina, 25 Courtenay Drive MSC 226, Charleston, SC 29425, USA; ^2^Department of Diagnostic and Interventional Radiology, Johann Wolfgang Goethe University Hospital, 60590 Frankfurt am Main, Germany; ^3^Department of Radiological Sciences, Oncology and Pathology, University of Rome “Sapienza”-Polo Pontino, Latina, Italy; ^4^Institute for Clinical Radiology, Ludwig-Maximilians-University Hospital, Marchioninistr. 15, 81377 Munich, Germany; ^5^Department of Radiology, Beijing Anzhen Hospital, Capital Medical University, Beijing 100029, China; ^6^Heidelberg Kidney Center, Department of Nephrology, University Hospital Heidelberg, INF 162, 69120 Heidelberg, Germany

## Abstract

The exact pathophysiology of contrast-induced nephropathy (CIN) is not fully clarified, yet the osmotic characteristics of contrast media (CM) have been a significant focus in many investigations of CIN. Osmotic effects of CM specific to the kidney include transient decreases in blood flow, filtration fraction, and glomerular filtration rate. Potentially significant secondary effects include an osmotically induced diuresis with a concomitant dehydrating effect. Clinical experiences that have compared the occurrence of CIN between the various classes of CM based on osmolality have suggested a much less than anticipated advantage, if any, with a lower osmolality. Recent animal experiments actually suggest that induction of a mild osmotic diuresis in association with iso-osmolar agents tends to offset potentially deleterious renal effects of high viscosity-mediated intratubular CM stagnation.

## 1. Introduction

Although osmotic characteristics of contrast agents in current use have been a significant focus in both animal and human studies [1, 2], the exact pathophysiology of contrast-induced nephropathy (CIN) is still not fully clarified [3–5]. High osmolality of water soluble contrast media (CM) has been shown to be responsible for significant hemodynamic, cardiac, and subjective effects including vasodilatation, heat, pain [6], and a variety of rheological effects such as red blood cell crenation [7] and renal osmotic diuresis [1, 6]. Universal implementation of lower osmotic, nonionic contrast agents has significantly reduced serious acute systemic side effects [6] making these drugs among the safest in medical use today. Yet, the major driving forces in the evolution of CM use today have been to eliminate systemic reactions and neurotoxicity [8], including disruption of the blood brain barrier [9] rather than renal toxicity. It has been presupposed that osmotoxicity is fundamental to CIN. This review will critically examine what is known about the role of CM osmolality in CIN.

## 2. Classification of Contrast Materials Based on Osmolality 

Currently available iodinated contrast agents are based on one (monomers) or two (dimers) tri-iodinated benzene rings, which can be broadly classified into three groups according to their osmolality, defined as the number of particles dissolved in one kilogram of water [2]. High osmolar contrast media (HOCM) are 5 to 8 times the osmolality of blood, (1,500 to over 2,000 mOsm/kg H_2_O) among which are older formulations that became available in the 1950s. Newer agents, although still more than three times the osmolality of blood, are of relatively low osmolality and are termed “low osmolar contrast media” (LOCM; up to 900 mOsm/kg H_2_O). All but ioxaglate are nonionic. These contrast agents include (in alphabetical order): iobitridol (XENETIX, Guerbet, France), iohexol (OMNIPAQUE, GE Healthcare, USA), iomeprol (IOMERON, Bracco Diagnostics, USA), iopamidol (ISOVUE, Bracco Diagnostics, USA), iopromide (ULTRA-VIST, Bayer HealthCare Pharmaceuticals, Germany), ioversol (OPTIRAY, Covidien, USA), ioxaglate (HEXABRIX, Guerbet, France), and ioxilan (OXILAN, Guerbet, France). Iodixanol (Visipaque, GE Healthcare), the only nonionic dimer in general clinical use today, was introduced in the mid-1990's and represents an iso-osmolar contrast medium (IOCM). As such, its osmolality is equal to that of blood (290 mOsm/kg H_2_O).

## 3. Effects of Contrast Medium Osmolality on Renal Physiology

A widely proposed mechanism for CIN is decreased renal blood flow [4]. Following CM injection into the main renal artery, renal blood flow (RBF) shows a biphasic response [18–20], that is an initial increase followed by prolonged reduction [18]. Reduced blood flow after CM injection is unique to the kidney as systemic vascular responses to CM in all other vascular beds are marked by vasodilation [2, 20, 21]. Therefore, renal ischemia has been thought to be a primary factor in the pathophysiology of CIN. Pharmacologic blockade of endogenous renal vasoconstrictors has failed to eliminate the decreased RBF in the experimental setting. Conversely, mannitol matched for CM osmolality and hypertonic saline solution both produce renal hemodynamic effects similar to those observed with administration of CM [22–30]. A reduction in both filtration fraction (FF) and glomerular filtration rate (GFR) has also been observed simultaneously with the decrease in RBF after CM administration. In contradistinction renal vasoconstrictors such as norepinephrine, angiotensin II, and serotonin produce an increase in FF with the expected decrease of RBF and GFR [18].

The induced reduction of GFR by osmotic CM can be explained by intratubular pressure changes. The osmotic forces, associated with CM molecules undergoing renal filtration, increase osmotic pressure in the proximal tubules and Bowman's capsule which by Starlings Law results in a lowered hydrostatic filtration pressure gradient across the filtering membrane of the glomerulus [1]. Due to their lowered osmolality, LOCM have been shown to minimize these effects [1, 2, 31–34].

## 4. Vacuolization in the Proximal Tubular Cell Cytoplasm

HOCM have been linked to an observation termed “osmotic nephrosis”—the induction of vacuolization in the cytoplasm of the renal proximal tubular cells. This phenomenon is attributed to the osmolality of the CM since similar osmotic diuretics have also been shown to produce these findings. These vacuolizations have been reported to occur more commonly in patients with preexisting renal insufficiency and are nonspecific but may rarely be associated with acute kidney injury (AKI) [35]. There is no proven link between vacuolization and CIN. Proximal tubular cell vacuoles have also been recently noted to occur with the IOCM, iodixanol, and have been associated with prolonged renal nephrograms demonstrated by CT [36]. Carraro et al. have suggested that it is the maximal urinary iodine concentration, rather than osmolality, that is responsible for the vacuolization in the proximal tubules occurring after iodixanol administration [37, 38].

## 5. Prerenal Effects of Hyperosmolar Contrast Media

All iodinated CM are osmotic diuretics; however, higher osmolality CM elicit greater diuresis. As such, patients will undergo an osmotic diuresis when given CM and as a consequence experience a dehydrating effect. This is of greater clinical concern if large doses of CM are administered over a short period of time in populations with multiple, closely spaced CT scans or extended cardiac catheterization procedures. It is quite possible that incidences of CIN could be the result of this nonspecific side effect, rather than a direct renal toxicity of the CM, per se. This may also help to explain the well-known beneficial effects of prophylactic hydration for CIN.

## 6. Comparison of the Incidence of CIN with Differing Osmotic Classes of Contrast Media

Head-to-head clinical comparisons between the different osmotic classes of CM have been performed comparing their effects on renal function. CIN is currently defined for these trials by an increase in serum creatinine concentration (SCr) within the first 5 days subsequent to contrast medium administration as a surrogate of decreased kidney function [39]. HOCM have been compared to LOCM and LOCM have been compared against IOCM (iodixanol) for both intravenous and intra-arterial administration. Here we provide an overview of relevant clinical reports.

### 6.1. Clinical Studies of HOCM versus LOCM for CIN

Surprisingly, comparisons between HOCM and LOCM have shown a much less than anticipated advantage for the ability of LOCM to decrease the risk of CIN, even in subjects with preexisting renal impairment.

In one large randomized double blinded study, Moore et al. included 929 patients receiving either HOCM or LOCM when undergoing diagnostic angiocardiography or contrast enhanced body CT [40]. CIN was defined as a 33% or 0.4 mg/dL increase in SCr from baseline where comparative creatinine levels were measured 48 hours after intervention. There was no overall difference in CIN between HOCM and LOCM. Only a “marginal difference” (*P* < 0.06) in CIN between HOCM and LOCM was found in subjects with preexisting renal insufficiency (RI), (SCr > 1.5 mg/dL). Other factors identified that rendered these patients at higher risk were angiocardiographic examinations, insulin-dependent diabetes, and the utilization of furosemide.

Drawing from a pool of 45 primary trials, Barrett and Carlisle (1993) conducted a large meta-analysis of 25 clinical trials with data of patients with increased SCr > 0.5 mg/dL after administration of CM [41]. They found no advantage of LOCM over HOCM in the setting of CIN with I.V. administration. Overall, pooling data for both I.V. and I.A. studies, there was only a small difference for CIN after comparing LOCM to HCOM. The presence of precontrast RI was the only factor identified that influenced the difference between the media effects. The results were reported to be “encouraging” that LOCM “may obviate more severe renal injury in patients with renal impairment,” but the authors did not go so far as to recommend the routine use of LOCM in patients with normal renal function.

Specifically for I.A. use and the occurrence of CIN between the LOCM iopamidol and the HOCM sodium diatrizoate, Schwab et al. [42] were unable to demonstrate a difference in a randomized controlled trial of 443 patients undergoing cardiac catheterization of whom 160 (36%) were at high risk (diabetes mellitus, heart failure, or preexisting renal failure (baseline SCr > 133 *μ*mol/L)). Additionally, Rudnick et al. [43] found no difference in CIN in low risk patients, even if diabetes was present in their randomized, double-blind, multicenter study of 1,196 patients. However, “high risk patients” with elevated creatinine at baseline alone, or combined with diabetes, had a 3.3 times higher rate of CIN with the HOCM sodium diatrizoate, compared to the LOCM iohexol. There was no difference between HOCM and LOCM in the incidence of severe adverse renal events, though the total number of cases was small (15 cases in total, 8 of which required acute dialysis; 5 with iohexol; and 3 with diatrizoate).

In summary, these studies do not convincingly demonstrate the decrease in risk for CIN between HOCM and LOCM that was anticipated for lowered osmolality CM. Additionally the highlighted studies did not include a control group for reference of the incidence of CIN, as some later studies would, an important consideration towards the validity of assessment based on SCr measurements [44].

### 6.2. Clinical Studies Comparing LOCM and IOCM for I.A. Use with an Emphasis on Meta-Analyses

In this section, studies were distinguished by route of administration as this appears to be a considerable source of confounding bias [45]. Intra-arterial administration has generally been associated with a higher risk of CIN. Potential factors contributing to discrepant incidences are higher underlying morbidity in patient populations clinically indicated to receive procedures that require I.A. CM administration, as well as procedural trauma and associated complications. Consequently trials recording the incidence of CIN occurring with I.A. injection likely overestimate the risk for patients receiving IV administration of CM for diagnostic imaging [44, 45].

A highly influential clinical report that compared the LOCM iohexol to the IOCM iodixanol for CIN was the NEPHRIC study [46]. The trial was set up in a double blind, prospective, multicenter design including 129 patients presenting with creatinine levels between 1.3 and 3.5 mg/dL, diabetes mellitus, and clinical indication for invasive catheter angiography. The incidence of CIN, defined by an absolute SCr elevation of 0.5 mg/dL, was 3% in the iodixanol group and 26% in the iohexol group (which was exceptionally high when compared to later studies). Peak increase of SCr in the 3 days following CM administration was also significantly smaller in the iodixanol group (iodixanol: 0.13 mg/dL; iohexol: 0.55 mg/dL; *P* = 0.001). Intra-arterial administration, selection of small patient cohorts, and specific CM gave little generalizability to these results. However, the findings stimulated numerous subsequent studies. Our attention is turned to ensuing meta-analyses that have attempted an all-encompassing assembly and appraisal of data pertinent to the question of LOCM versus IOCM for I.A. administration and the occurrence of CIN. We highlight these studies because of their high impact which when combined can provide a comprehensive overview.

The first meta-analysis was performed by Solomon et al. in 2005 [10] and reviewed seventeen primary studies from 1991 to 2004 with a total of 1,365 patients [10] from a selection of 1594 citations (Table 1). Six nonionic LOCM agents as well as iodixanol were administered intra-arterially in these studies. Between iodixanol and iopamidol the risk of CIN was found to be similar with both of these agents having a significantly lower risk for CIN than iohexol. A significant advantage for iodixanol in this analysis was only shown in direct comparison with iohexol. In individual comparison iohexol also resulted in a higher incidence of CIN when compared to all other low osmolality agents while iopamidol was associated with a lower incidence of CIN compared to all other agents. From these results the authors concluded that osmolality alone cannot account for the observed differences in CIN.

This study was followed by McCullough et al. [11] with an analysis from the iodixanol database (GE Healthcare) pooling individual data of 2,727 patients from 16 double-blinded, randomized, controlled trials using I.A. administration between 1991 and 2003. Results from I.A. administration showed a benefit of iodixanol (*n* = 1, 382) compared to the pooled LOCM population (*n* = 1, 345) composed of iohexol and ioxaglate in the vast majority of cases. The study further demonstrated a lower incidence of CIN, by absolute threshold of 0.5 mg/dL from baseline, with iodixanol use (2.4%, versus 6.2%; *P* = 0.002) even before stratifying for elevated baseline SCr and presence of diabetes mellitus. The authors therefore concluded lower risk for CIN with iodixanol than LOCM in all cases, and particularly in patients with elevated baseline SCr or elevated baseline SCr and diabetes mellitus.

Further expanding the scope of pooled data, Heinrich et al. [12] published a meta-analysis in 2009 including 25 randomized controlled trials in the timeframe from 1950 to 2007 from a selection of 926 trials, pooling data of 3,270 patients. Contrast media included several nonionic LOCM and iodixanol. For intra-arterial use in patients with prior renal insufficiency and diabetes mellitus, there was no decrease of CIN incidence associated with iodixanol compared with LOCM other than iohexol [12]. Independent from route of administration or preexisting renal insufficiency, the use of iohexol versus LOCM other than iohexol had a significant influence on the relative risk of CIN (*P* < 0.01). The authors stated in conclusion that evidence does not suggest iodixanol to be less nephrotoxic than LOCM, with the exception of iohexol in the case of intra-arterial administration and prior renal insufficiency.

A more recent analysis by From et al. [13] in 2010 included 36 randomized controlled trials from an initial selection of 112 abstracts arriving at a total of 7166 patients (3672 patients received iodixanol and 3494 patients received other LOCMs) between 1966 and 2009. Differences in incidence of CIN between iodixanol and the pooled LOCM did not reach statistical significance [13]. Overall, a significant reduction of CIN with iodixanol was only shown in direct comparison of iodixanol with iohexol. Even in subanalysis of patients receiving intra-arterial CM, there was no benefit for iodixanol over LOCM other than in individual comparison to iohexol. The authors further point out that this significance is attributable to inclusion of one study [46], where reported incidences of CIN are higher than those seen in any other trial arm of the studies included.

These studies present supportive evidence that osmolality is not the decisive factor for the incidence of CIN at osmolality levels of LOCM or IOCM, even when used intra-arterially [41]. Apparent differences in recorded nephrotoxicity between agents of similar osmolality (iohexol and iopamidol) hint towards other contributing factors, such as direct tubular toxicity as investigated by prior animal studies [10, 47–49]. Differences between iohexol and other LOCM would most likely be a consequence of the specific molecular structure as there is no other known pathogenetic mechanism.

### 6.3. Clinical Studies of LOCM versus IOCM for I.V. (CT) Use

Of particular clinical relevance is the evaluation of intravenous CM administration for contrast enhanced CT studies [50, 51]. Amongst four comparable head-to-head prospective studies published between 2006 and 2008 comparing intravenous iodixanol and LOCM in patients with renal insufficiency, two found no significant difference [14, 15], one suggested a lower incidence of CIN with iodixanol (over iopromide) [17], and one found a higher incidence of CIN with iodixanol (over iomeprol) [16] (Table 2).

Barrett et al. [14] included 153 patients with elevated baseline SCr of which 36 had diabetes mellitus. Both groups were comparable in distribution of age, gender presence of diabetes, hydration, and concomitant medication. Of the 77 patients receiving iopamidol (370 mgI/dL), in equal total iodine-dose to the iodixanol arm, none showed an absolute increase of >0.5 mg/dL SCr. In the iodixanol group a small number of patients showed such an increase (2 of 76 patients, 2.6%; *P* = 0.2). A relative increase in SCr, above 25% of baseline measurement, occurred in equal relative distribution (4%) among both study arms (95% confidence interval −6.2-6.1, *P* = 1.0).

Using the same contrast agents, Kuhn et al. [15] enrolled only patients who presented with both diabetes mellitus as well as elevated baseline SCr in their study. A total of 258 patients were randomized to receive either iopamidol (370 mgI/dL) or iodixanol (320 mgI/dL). A significantly higher total iodine dose was present in the iopamidol arm after comparable volumes of both agents were administered. Mean serum creatinine change from baseline was equal in both groups (0.04 mg/dL). The incidence of CIN by relative increase from baseline SCr was similarly low in both arms: 5.6% for iopamidol and 4.9% for iodixanol (95% CI, −4.8% to 6.3%; *P* = 1.0). There was no statistical difference for the incidence of CIN between the two classes of agents. It was concluded that there was no difference in the incidence of CIN as shown within this high risk population between iopamidol and iodixanol.

Thomsen et al. [16] investigated iomeprol-400 (400 mgI/mL, 726 mOsm/kg) in the LOCM cohort of this multicenter trial. The authors evaluated 76 of 148 patients with equivalent iodine-dose as in the iodixanol-320 cohort (320 mgI/mL, 290 mOsm/kg). CIN, again defined by relative elevation from baseline (above 25% SCr baseline), did not differ significantly (*P* > 0.05) in the study group. Absolute elevation of SCr above 0.5 mg/dL was observed in the iodixanol-320 group only (in 5 of 72 patients). This difference reached statistical significance (*P* = 0.025). Mean SCr changes from baseline in both groups also differed significantly: 0.06 ± 0.27 mg/dL with iodixanol-320 and −0.04 ± 0.19 mg/dL with iomeprol-400. Thus, the authors concluded that in patients with moderate to severe chronic kidney disease the incidence of CIN is higher after intravenous administration of iodixanol than iomeprol.

Using the same criteria of elevated baseline SCr, Nguyen et al. [17] enrolled 126 patients between 2004 and 2006. Iodixanol-320 (61 patients) was compared to iopromide-370 (370 mgI/mL; Ultravist, Bayer) (56 patients). The incidence of CIN by relative SCr increase of 25% from baseline was 8.5% (5 of 61) for iodixanol and 27.8% (15 of 56) for iopromide (*P* = 0.012). The incidence of CIN by absolute SCr elevation of more than 0.5 mg/dL was observed in 5.1% (3 of 61) in the iodixanol group and 18.5% (10 of 56) in the iopromide group (*P* = 0.037). Significant reduction of CIN incidence was thus reported with iodixanol compared to iopromide.

Observation of background fluctuation of SCr measurements was possible in a large retrospective nonrandomized analysis comparing iohexol and iodixanol by Bruce et al. [52]. This trial also included a control group of patients receiving unenhanced CT. Within all groups, 11,588 patients were included between 2000 and 2006. While the iohexol-group contained 5,328 patients, the iodixanol group was considerably smaller containing 462 patients. The remaining 7,484 patients received no CM. The authors found no significant difference in the overall incidence of CIN between the IOCM iodixanol (8.2%) and control groups (5.9%) for all baseline creatinine values. The overall incidence of CIN in the LOCM group paralleled that of the control group up to a SCr level of 1.8 mg/dL. Increases in SCr above this level were associated with a higher incidence of CIN in the LOCM group. We consider this to be an important study, however of somewhat less significance than the above-noted head-to-head prospective studies for the following limitations. First, patients were not assigned prospectively but treated according to current CM protocols. Second, considerable differences in demographics existed and no propensity scoring was performed to reconcile this. Overall no additional incidence of CIN was found with iodixanol use in comparison to the control group. A disadvantageous comparison of iohexol to iodixanol was confirmed in high risk patients. The authors therefore suggested use of iodixanol over iohexol in this high risk group. In conclusion, the high rate of CIN within the control group of this study introduces further problems as to the validity of studies assessing the incidence of CIN after CM administration without control groups.

From the studies presented, the risk of CIN shows no consistent difference between LOCM and IOCM. Individual comparisons suggest a higher risk for iohexol than other LOCM when compared to IOCM. Furthermore, equal incidence of risk for CIN with iopamidol and lower incidence of risk with iomeprol over iodixanol have been reported. These discrepancies illustrate that risk stratification based on osmolality alone is not sufficient. Further salient factors and limitations in measurements have been repeatedly put forward.

## 7. Shifting Paradigm That Osmolality May Actually Be Beneficial

Recent attention has turned to CM viscosity being a more important contributing factor in the pathophysiology of CIN. Iodixanol is the newest iodinated contrast agent to be developed, having an iso-osmolar profile, but with a much higher viscosity than the low osmolar monomers. In animal studies viscosity-mediated decreases in GFR, urine flow and renal medullary blood flow have been demonstrated [4, 53, 54]. Viscosity increases of up to 50-fold have been shown for the iso-osmolality agent iodixanol [55]. Urinary fluid viscosity with iodixanol is markedly higher than LOCM at equal iodine concentrations [56]. Initial differences become even more prominent as the filtrate becomes more concentrated along the tubular system of the kidney, since CM viscosity increases exponentially rather than linearly with higher concentrations [57]. Increased viscosity creates urinary stagnation, increased hydrostatic pressure in Bowman's space, and by Starlings law a decreased hydrostatic gradient for glomerular capillary filtration. Medullary blood flow is also diminished with increasing capillary fluid viscosity [58]. Increased red blood cell aggregation is reported to be a characteristic of the iso-osmotic dimers [59]. Viscosity has therefore been highlighted as a significant factor amongst the scope of chemical properties of iodinated contrast agents suspected to impede renal function, [60].

One strategy to diminish the viscosity effect of the iso-osmotic dimers is by enhancing tubular flow with a concomitant osmotic diuresis, as occurred naturally with the LOCM [36, 61]. Lenhard et al. [36] generated an iodixanol/mannitol formulation with a similar osmolality to the LOCM iopromide in a rat model and eliminated the enhanced expression of kidney injury markers caused by iodixanol-only injection. An important assumption here is that prolonged iodine exposure in the kidney is harmful [62]. Indeed, Liss et al. [63] have shown a higher rate of CIN with an IOCM, iodixanol, than with LOCM in a large retrospective study of 57,925 patients undergoing coronary procedures in Scandinavia.

## 8. Conclusion

From the preceding there is no conclusive evidence that osmolality, within the range that includes LOCM and IOCM, is the prominent factor for CIN. Furthermore, suggestions have been proposed that osmolality levels, within the range of currently available LOCM, might actually have nephroprotective effects.

## Figures and Tables

**Table 1:** Meta-analyses for CIN comparison of LOCM* versus IOCM** (mainly I.A.).

Authors (date)	Search results	Analyzed trials	Total patients	Dates included	Agent superiority
Solomon (2005), [10]	1,594	17	1,365	1991–2004	LOCM = IOCM > Iohexol
McCullough et al. (2006), [11]	Iohexol database	16	2,727	1991–2003	IOCM > LOCM
Heinrich et al. (2009), [12]	926	25	3,270	1950–2007	LOCM = IOCM > Iohexol
From et al. (2010), [13]	112 (abstracts)	36	7,166	1966–2009	IOCM ≥ LOCM

*LOCM: low osmolality contrast media.

**IOCM: iso-osmolar contrast media.

**Table 2:** Clinical studies for CIN comparison of LOCM* versus IOCM** (mainly I.V.).

Authors (date)	Patients evaluated (originally included)	CIN absolut^1^ (relative^2^) LOCM	CIN absolut^1^ (relative^2^) IOCM	Agent superiority
Barrett et al. (2006), [14]	153 (166)	0/77 (3/77)	2/76 (3/76)	LOCM = IOCM

Kuhn et al. (2008), [15]	248 (264)	(6/123)	(7/125)	LOCM = IOCM

Thomsen et al. (2008), [16]	148 (184)	0/76 (4/76)	5/72 (5/72)	LOCM > IOCM

Nguyen et al. (2008), [17]	126 (117)	10/56 (15/56)	3/61 (5/61)	IOCM > LOCM

*LOCM: low osmolality contrast media.

**IOCM: iso-osmolar contrast media.

SCr: serum creatinine.

CIN: contrast-medium induced nephropathy.

^1^SCr increase ≥0.5 mg/dL.

^2^SCr increase ≥25%.

## Abbreviations

AKI: Acute kidney injury
CIN: Contrast-medium induced nephropathy
CKD: Chronic kidney disease
CM: Contrast medium
CT: Computed Tomography
FF: Filtration fraction
GFR: Glomerular filtration rate
HOCM: High osmolar contrast medium
IOCM: Iso-osmolar contrast medium
LOCM: Low osmolar contrast medium
RBF: Renal blood flow
RI: Renal insufficiency
SCr: Serum creatinine concentration.
